# Estradiol-induced senescence of hypothalamic astrocytes contributes to aging-related reproductive function declines in female mice

**DOI:** 10.18632/aging.103008

**Published:** 2020-04-07

**Authors:** Xiaoman Dai, Luyan Hong, Hui Shen, Qiang Du, Qinyong Ye, Xiaochun Chen, Jing Zhang

**Affiliations:** 1 Department of Neurology and Geriatrics, Fujian Institute of Geriatrics, Fujian Medical University Union Hospital, Fujian Key Laboratory of Molecular Neurology, School of Basic Medical Sciences, Fujian Medical University, Fuzhou 350001, Fujian, China; 2 Department of Hepatobiliary Surgery and Fujian Institute of Hepatobiliary Surgery, Fujian Medical University Union Hospital, Fuzhou 350001, Fujian, China; 3 Department of Biochemistry and Molecular Biology, Gannan Medical University, Ganzhou 341000, Jiangxi, China

**Keywords:** estradiol, aging, hypothalamic astrocyte, reproduction, GnRH

## Abstract

Hypothalamic astrocytes are important contributors that activate gonadotropin-releasing hormone (GnRH) neurons and promote GnRH/LH (luteinizing hormone) surge. However, the potential roles and mechanisms of astrocytes during the early reproductive decline remain obscure. The current study reported that, in intact middle-aged female mice, astrocytes within the hypothalamic RP3V accumulated senescence-related markers with increasing age. It employed an ovariectomized animal model and a cell model receiving estrogen intervention to confirm the estrogen-induced senescence of hypothalamic astrocytes. It found that estrogen metabolites may be an important factor for the estrogen-induced astrocyte senescence. In vitro molecular analysis revealed that ovarian estradiol activated PKA and up-regulated CYPs expression, metabolizing estradiol into 2-OHE_2_ and 4-OHE_2_. Of note, in middle-aged mice, the progesterone synthesis and the ability to promote GnRH release were significantly reduced. Besides, the expression of growth factors decreased and the mRNA levels of proinflammatory cytokines significantly increased in the aging astrocytes. The findings confirm that ovarian estradiol induces the senescence of hypothalamic astrocytes and that the senescent astrocytes compromise the regulation of progesterone synthesis and GnRH secretion, which may contribute to the aging-related declines in female reproductive function.

## INTRODUCTION

Age-related female reproductive decline is associated with the neuroendocrine regulation of the hypothalamic-pituitary-ovarian axis (HPO), in which an attenuated luteinizing hormone (LH) surge is one of the earliest signs associated with reproductive senescence in female rats and mice [1, 2]. Previous research has documented that the activation of gonadotropin-releasing hormone (GnRH) neurons is remarkedly reduced in middle-aged mice during the GnRH/LH surge [3–5]. However, the underlying mechanisms of the hypothalamic regulation for the early reproductive decline remain obscure.

To date, a body of studies have documented that hypothalamic astrocytes are one of the key cells that constitute the neural circuits responsible for GnRH neuron activation and GnRH release. Studies in rodents have shown that ovarian estradiol (E_2_) stimulates progesterone synthesis in hypothalamic astrocytes, which is the key to the activation of the GnRH neurons [6–8]. Specifically, estradiol acts on membrane-associated estradiol receptor-alpha (ERα) in astrocytes to increase the release of intracellular store of Ca^2+^ via a phospholipase C (PLC)/ inositol trisphosphate (IP3) signaling pathway. The increased Ca^2+^ level in turn activates the protein kinase A (PKA), leading to protein phosphorylation and the activation of cholesterol-side-chain cleavage enzyme (P450scc) and 3β-hydroxysteroid dehydrogenase (3β-HSD), which are responsible for limiting the rate of progesterone synthesis. Then, the newly synthesized progesterone then diffuses out of the astrocytes and activates estradiol-induced progesterone receptors in proximal neurons, triggering the neural cascades and resulting in the GnRH/LH surge [6].

Available studies have also demonstrated that astrocytes are essential for the GnRH neuronal activity and GnRH release through multiple pathways. In rodents and rhesus monkeys, the remarkable structural plasticity of astrocytes has been confirmed to ensheath GnRH cell bodies and/or to oppose the GnRH processes. These structural relationships are dynamically regulated by ovarian estradiol and are crucial for normal female reproductive function [9–11]. In addition, astrocytes also synthesize and release cytokines that regulate GnRH secretion, including prostaglandin E and a lot of growth factors, such as transforming growth factors (TGFα and TGF-β1), basic fibroblast growth factor IGF1, and epidermal growth factor family, etc. [12–15].

Taken together, the existent literature shows that astrocytes play a crucial part in the puberty onset, estrus cycle, and fertility [7, 8, 16]. But what changes take place in the hypothalamic astrocytes during aging remains blurred. Therefore, we hypothesized that when the repeated cycles of ovarian estradiol induce the synthesis of neuroprogesterone in hypothalamic astrocytes, the metabolic pathway of estradiol also simultaneously accelerates the senescence of astrocytes, which would weaken or change the role of astrocytes in regulating the female reproductive function. We tested this hypothesis with a mouse model receiving bilateral ovariectomy and an estradiol replacement and in vitro primary cultured hypothalamic astrocytes treated with estradiol.

## RESULTS

### Astrocytes within the hypothalamic RP3V accumulate senescence-related markers with increasing age

**Figure 1:**
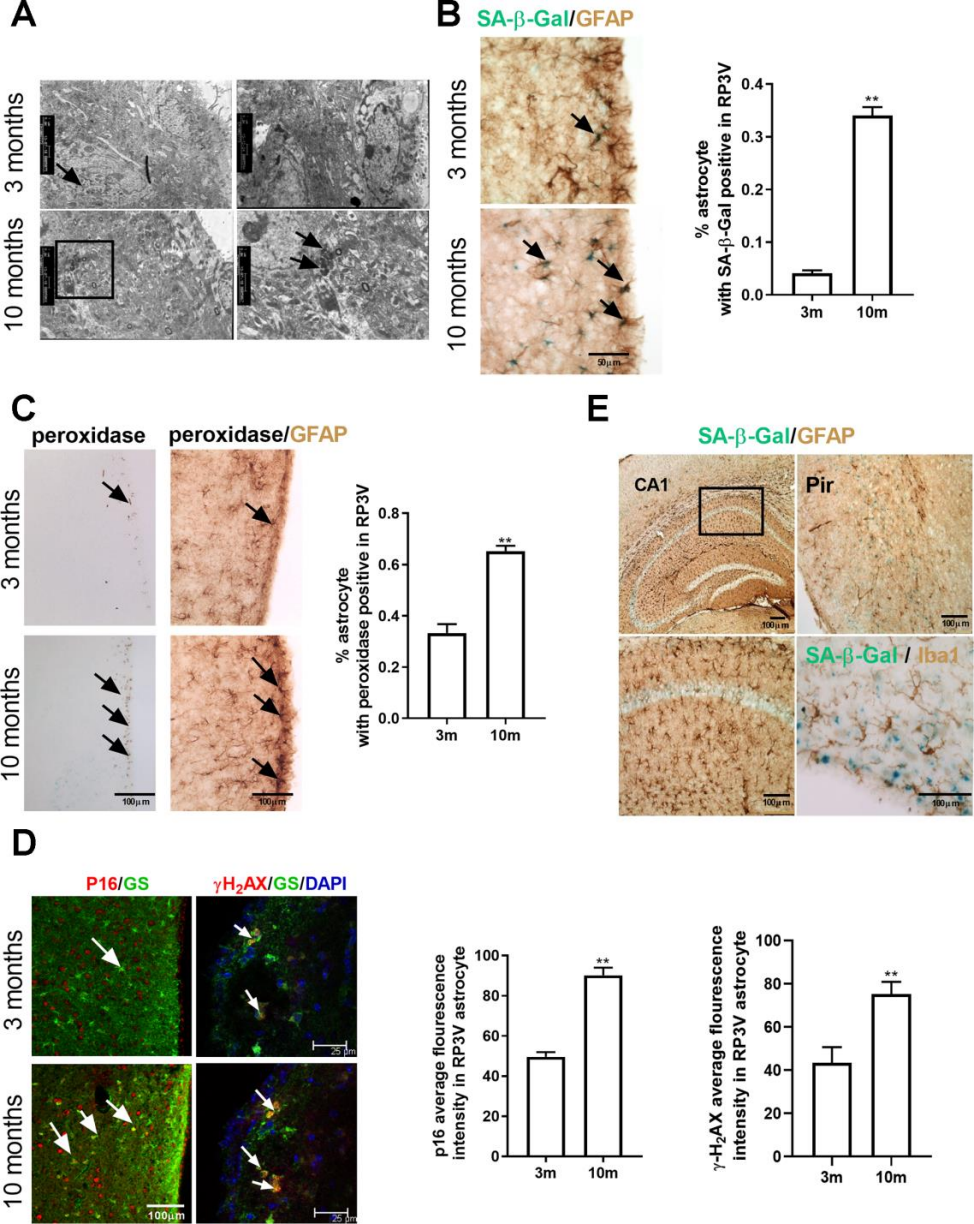
**Astrocytes within the hypothalamic RP3V accumulates senescence-related markers with increasing age.** (**A**) The lipofuscin deposition by transmission electron microscopy in hypothalamic astrocytes of female C57BL/6J mice at the age of 3 months and 10 months. Black arrows represent the lipofuscin deposition. (**B**) Dual-label immunohistochemistry of astrocytes by GFAP staining (brown) and by SA-β-Gal staining (blue) in 3-month-old mice (n=5) and 10-month-old mice (n=5), black arrows representing SA-β-Gal –positive astrocytes, scale bar=50μm. (**C**) Peroxidase staining (brown) in the astrocytes of RP3V (left), black arrows representing peroxidase. GFAP (black) and peroxidase (brown) double staining in astrocytes of RP3V in the hypothalamus of 3-month-old mice (n=5) and 10-month-old mice (n=5), black arrows representing peroxidase–positive astrocytes, scale bar =100μm. (**D**) Dual-label immunofluorescence showing astrocytes (green) with p16 (red) in young (n=5) and middle-aged mice (n=5), white arrows representing p16–positive astrocytes, scale bar=100μm (left). Dual-label immunofluorescence showing astrocytes (green) with γ-H2AX (red) in young (n=5) and middle-aged mice (n=5), white arrows representing γH2AX–positive astrocytes, scale bar=25μm (right). (**E**) Dual-label immunohistochemistry showing astrocytes (brown) with SA-β-Gal staining (blue) in 10-month-old mouse cortex (left picture) and hippocampal (top right picture). Dual-label immunohistochemistry showing microglia (brown) with SA-β-Gal staining (blue) in RP3V of 10-month-old mice (bottom right picture). Scale bar=100μm. The *p*-value was determined by Student’s t test,** *p*< 0.01.RP3V, i.e. rostral periventricular area of the third ventricle; GS, i.e. glutamine synthetase.

To observe the age-related changes of astrocytes in the rostral periventricular area of the third ventricle (RP3V) of the hypothalamus, the senescence-related markers of the astrocytes were compared between the young-aged and middle-aged group with regular estrous cycles. The senescence was evaluated by measuring the level of lipofuscin granules, a constant and reliable indicator of cell senescence [17]. Under an electron microscope, the number and volume of lipofuscin granules of hypothalamic astrocytes in the middle-aged group increased markedly (Figure 1A). A subsequent confirmation by senescence-associated β-galactosidase (SA-β-Gal) staining [18] combined with GFAP (astrocyte marker) immunohistochemistry reported an obviously higher percentage of SA-β-gal positive astrocytes in the middle-aged group than in young-aged mice(df=16; t=23.68; *p*<0.01) (Figure 1B). DAB staining was performed to detect the peroxidase activity and revealed an increased peroxidase activity in the middle-aged mice (Figure 1C). A further labeling of GFAP by immunohistochemistry showed a much higher percentage of peroxidase-positive astrocytes in the middle-aged mice than in the young ones (Unpaired t test, df=17; t=7.664; *p*<0.01)(Figure 1C). Finally, immunofluorescence was performed to detect in astrocytes the expression of p16 and γ-H_2_AX, which are the respective markers for senescence and DNA injury and are usually highly expressed in the senescent cells [19–21]. The analysis showed that compared with those of the young-aged mice, the mean fluorescence intensity of p16 and that of γ-H_2_AX in the astrocytes of the RP3V were both significantly enhanced in the middle-aged mice (Unpaired t test, df=30; t=9.44; *p*<0.01; df=8; t=3.492; *p*<0.01;respectively) (Figure 1D). These results show that astrocytes in the hypothalamic RP3V display age-related senescence.

To further confirm the type of senescent cells, Iba1 (a microglia marker) staining by immunohistochemistry was performed after the completion of SA-β-Gal staining, which found no SA-β-Gal positive microglia cells in the middle-aged mice (Figure 1E). To explore whether the senescence of astrocytes is selective in brain regions, SA-β-Gal staining was also performed on the hippocampal and cortical region of the middle-aged female C57BL/6J mice. The results showed no obvious senescent characteristics in the astrocytes of these two brain regions (Figure 1E). These findings suggest that the senescence of astrocytes in the middle-aged mice is selective in brain regions.

### Estradiol induces the senescence of astrocytes in the hypothalamus

**Figure 2:**
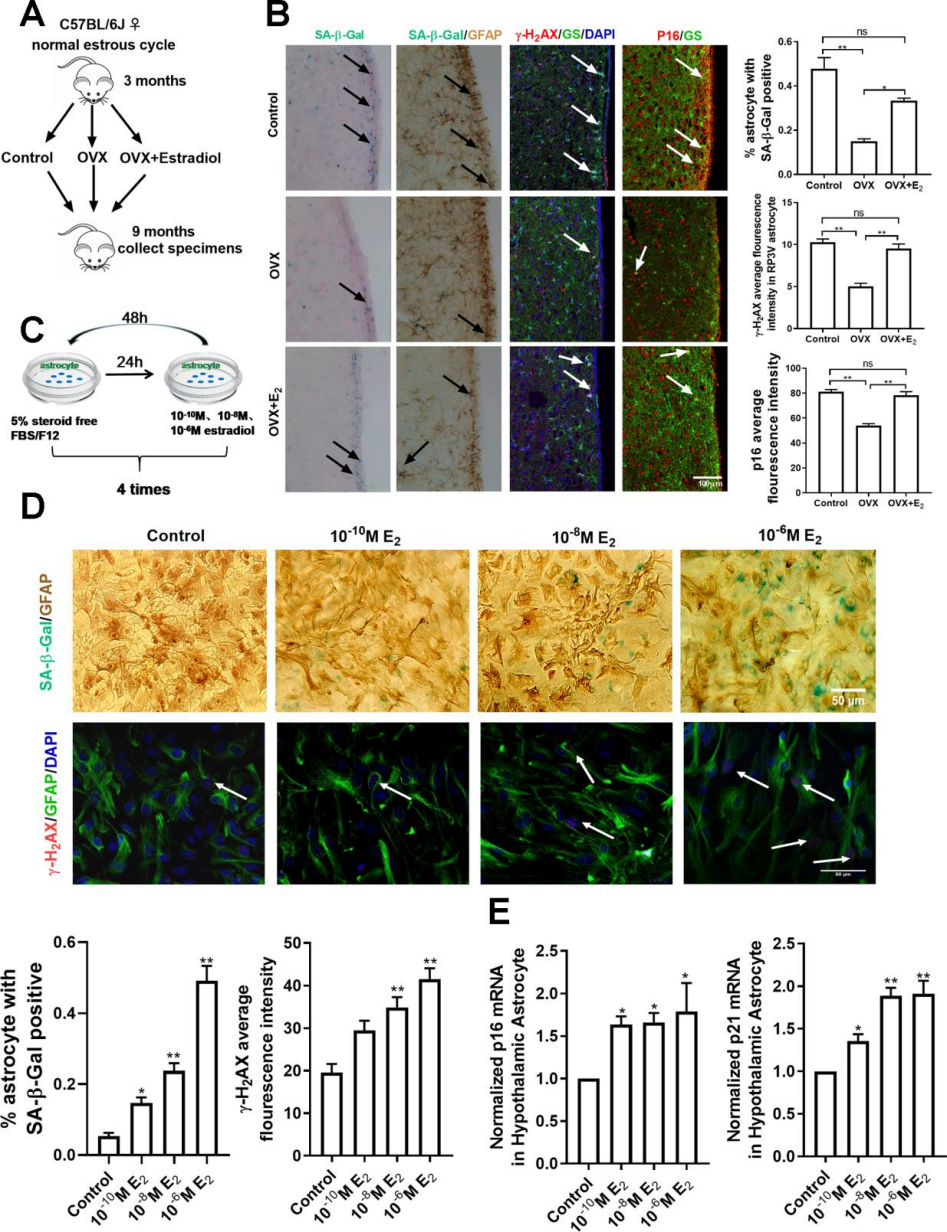
**Estradiol induces senescence of astrocytes in the hypothalamus.** (**A**) The flow chart of mouse castration and estradiol intervention. (**B**) Representative microscopies showing SA-β-gal staining in the control (n=5), OVX (n=5) and OVX+E_2_ groups (n=5), black arrows representing SA-β-Gal–positive cells (left one). Dual-label immunohistochemistry showing astrocytes by GFAP staining (brown) and by SA-β-Gal staining (blue), black arrows representing SA-β-Gal–positive astrocytes (left two). Dual-label immunofluorescence showing astrocytes (green) with γ-H2AX (red), white arrows representing γ-H2AX–positive astrocytes (right two). Dual-label immunofluorescence showing astrocytes (green) with p16 (red), white arrows representing p16–positive astrocytes (right one). Scale bar= 100μm. (**C**) The flow chart of estradiol intervention in primary cultured astrocytes. (**D**) Dual-label immunohistochemistry showing astrocytes by GFAP staining (brown) and by SA-β-Gal staining (blue) with three different estradiol concentrations (10^-10^M, 10^-8^M, 10^-6^M) (upper), black arrows representing SA-β-Gal–positive astrocytes. Scale bar= 100 μm. Dual-label immunofluorescence showing astrocytes (green) and γ-H2AX (red) with different estradiol concentrations, white arrows representing γ-H2AX–positive astrocytes. Scale bar=50μm. (**E**) Detection of *p16* and *p21* mRNA levels under different estradiol concentrations. Estradiol increased the expression of *p16* and *p21* with different estradiol concentrations in hypothalamic astrocytes (n = 3-4). The experiments used two-way analysis of variance. The *p*-value was determined by One-way ANOVA: *p<0.05, ** *p*< 0.01.OVX, i.e. ovariectomy, OVX+E_2_, i.e. ovariectomy plus estradiol replacement, 10^-6^M E_2_, i.e.10^-6^M estradiol concentrations.

As astrocytes in the hypothalamic RP3V play a crucial part in the estradiol positive feedback [16, 22, 23], it follows that the senescence of astrocytes in RP3V may be associated with ovarian estradiol. To confirm this hypothesis, a mouse model with ovarian resection was adopted in the study. A total of 45 female mice (aged 3 months old) with regular estrus cycles determined by daily vaginal smears were selected and randomly placed into 3 groups receiving bilateral ovariectomy (OVX group), bilateral ovariectomy plus estradiol replacement (OVX+E_2_ group) or sham-operation (control group). The OVX+E_2_ group received twice a 0.25mg dose of a 90-day sustained release pellet of 17β-estradiol placed subcutaneously. The dose of the 17β-estradiol in mice has been found to correspond to the estradiol levels in a pro-estrus phase [24, 25]. Brain tissue samples were obtained from the three groups when they were raised to 9 months old (Figure 2A). Compared with those of the sham group and OVX+E_2_ group, the number of SA-β-gal positive astrocytes in the RP3V of the OVX group was significantly reduced, and the mean fluorescence intensity of astrocytes expressing γ-H_2_AX and p16 was also obviously attenuated. Meanwhile, no significant difference was evident between the OVX+E_2_ and the sham group (one-way ANOVA effect of treatment *p*=0.0944, df=2,10, F=3.895; *p*=0.5026, df=2,81, F=0.4641; *p*=0.6268, df=2,42, F=1.614; respectively) (Figure 2B). The results show that the sustaining effect of estradiol is involved in the senescence of astrocytes in the hypothalamic RP3V.

To further confirm the above results, primary cultured astrocytes from the neonatal mouse hypothalamus were directly treated with 17β-estradiol. The intervention concentration of estradiol was 10^-6^M, 10^-8^M, and 10^-10^M, according to the experimental results of CCK8 (Supplementary Figure 1B). The astrocytes were first cultured with a carbon-adsorbed serum, which did not contain steroid hormones, for 24h before the 17β-estradiol intervention, and then incubated with different estradiol concentrations for 48 h. The process was repeated 4 times to induce cell senescence [6] (Figure 2C). The analysis showed that compared with the solvent-treated astrocytes, estradiol augmented the number of SA-β-Gal positive astrocytes in the hypothalamus in a concentration-dependent manner. The number of γ-H_2_AX positive astrocytes and the mean fluorescence intensity of γ-H_2_AX expression in astrocytes were significantly enhanced in the estradiol-treated group (10^-8^M and 10^-6^M) (one-way ANOVA effect of treatment *p*<0.05, df=3,11, F=1.219;Kruskal-Wallis test, *p*<0.01, respectively) (Figure 2D). In addition, the mRNA levels of *p16* and *p21* in the estradiol-treated group were significantly higher than those in the control sample (one-way ANOVA effect of treatment *p*<0.05, df=3,10, F=2.391;*p*<0.05, df=3,9, F=1.525, respectively) (Figure 2E). However, estradiol treatment did not induce a similar phenomenon in the cortex-derived astrocytes but displayed an opposite change instead, as shown in the supplemental materials (Supplementary Figure 1C–1E). Altogether, the results of in vivo and in vitro analyses show that estradiol induces the senescence of hypothalamic astrocytes.

### The estradiol metabolites 2-OHE_2_ and 4-OHE_2_ are closely associated with astrocyte senescence

**Figure 3:**
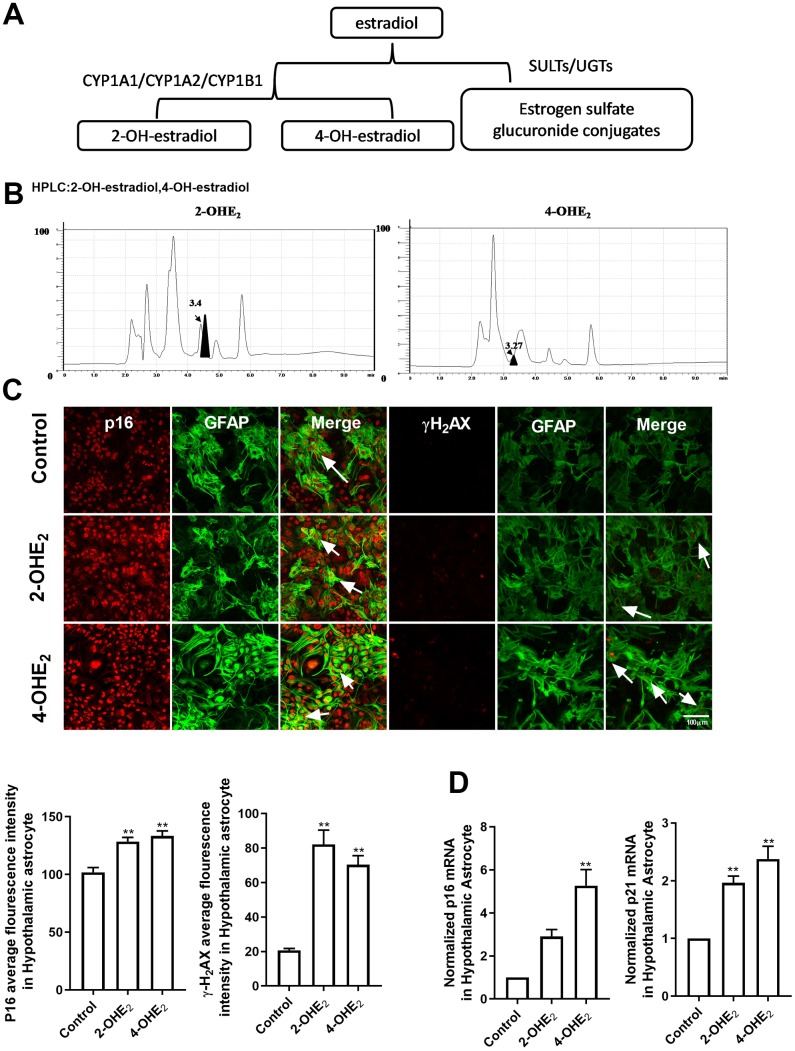
**The estradiol metabolites 2-OHE_2_ and 4-OHE_2_ are closely associated with astrocyte senescence.** (**A**) The flow chart of estradiol metabolism. (**B**) HPLC chromatogram of 2-OHE_2_ and 4-OHE_2_ in hypothalamic tissue of 10-month-old mice. (**C**) Dual-label immunofluorescence showing astrocytes (green) and p16 (red) with 2-OHE_2_ and 4-OHE_2_, white arrows representing γH2AX–positive astrocytes (left). Dual-label immunofluorescence showing astrocytes (green) and γ-H2AX (red) with 2-OHE_2_ and 4-OHE_2_, white arrows representing γ-H2AX–positive astrocytes (right). Scale bar= 100 μm. (**D**) Detection of *p16* and *p21* mRNA levels with 2-OHE_2_ and 4-OHE_2_ intervention. 2-OHE_2_ and 4-OHE_2_ respectively increased the expression of *p16* and *p21* in hypothalamic astrocytes (n=3). The *p*-value was determined by One-way ANOVA: ** *p*< 0.01.

To understand the mechanism of estradiol-induced cellular senescence, we investigated into the intracellular metabolic pathway of estradiol [26]. Estradiol metabolism operates in two pathways (Figure 3A): a conjugative pathway, which forms sulfates and glucuronides, respectively catalyzed by sulfotransferases (SULTs) and UDP-glucuronosyl transferases (UGTs), and a hydroxylated pathway. In the latter, estradiol is hydroxylated by *CYPs* (cytochrome p450) isoforms, including *CYP1A1*, *CYP1A2*, and *CYP1B1*, to form catechol estradiols, such as 2-hydroxyestradiol (2-OHE_2_) and 4-hydroxyestradiol (4-OHE_2_), which have long been thought to be an intermediate metabolite responsible for DNA damage and oxidative stress [27, 28]. So targeted HPLC was proceeded on hypothalamic tissues to examine the level of 2-OHE_2_ and 4-OHE_2_ and the results indicated their existence in the hypothalamus of the 10-month-old mice (Figure 3B). Furthermore, the primary cultured astrocytes from the hypothalamus were respectively treated with 2-OHE_2_ (20nM, 24h) and 4-OHE_2_ (20nM, 24h). The mean fluorescence intensity of p16 and γ-H_2_AX in the astrocytes treated with hydroxyl estradiols significantly increased when compared with that of the control group (one-way ANOVA effect of treatment *p*<0.01, df=2,96, F=0.5358;Kruskal-Wallis test, *p*<0.01, respectively) (Figure 3C). Similarly, the mRNA level of *p16* and *p21* in the astrocytes treated with hydroxyl estradiols was obviously higher than that of the control group (one-way ANOVA effect of treatment *p*<0.01, df=2,6, F=1.982; *p*<0.01, df=2,6, F=1.33, respectively) (Figure 3D). These results suggest that the intracellular metabolites of estradiol, 2-OHE_2_ and 4-OHE_2_, facilitate the astrocyte senescence.

### PKA-CYP signaling mediates estradiol-induced senescence of hypothalamic astrocytes

**Figure 4:**
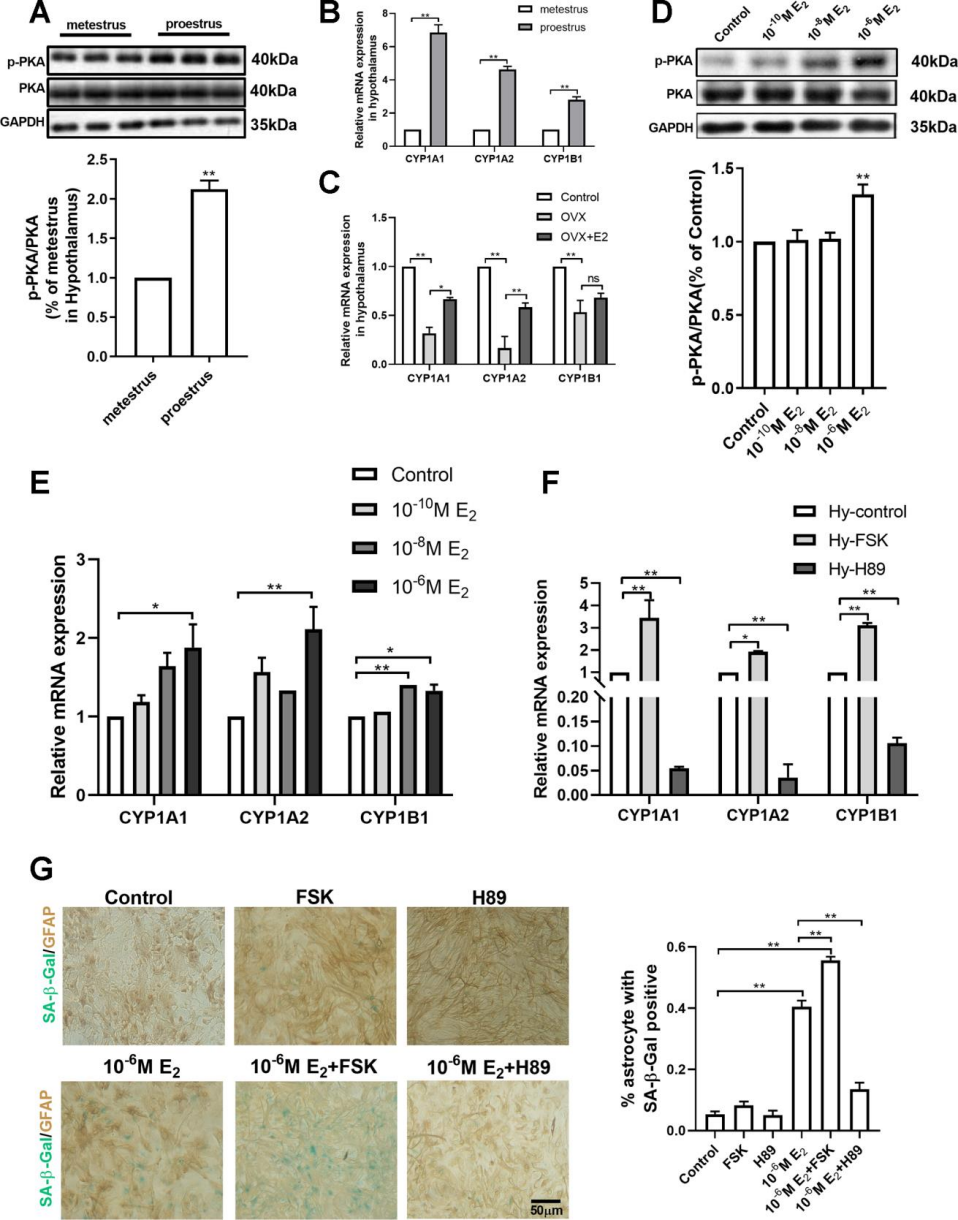
**PKA-CYP signaling mediates estradiol-induced senescence of hypothalamic astrocytes.** (**A**) The expression of PKA and p-PKA in hypothalamus during proestrus and metestrus at 3 months of age as determined by Western blotting (n = 6). (**B**) Effects of estrous cycle on the mRNA expression of *CYP1A1*, *CYP1A2* and *CYP1B1* gene in the hypothalamus as determined by qPCR. Metestrus vs. proestrus, n=3 (upper). (**C**) Effects of estradiol on the mRNA expression of *CYP1A1*, *CYP1A2* and *CYP1B1* gene in the hypothalamic tissue as determined by qPCR. OVX group vs. control group, OVX group vs. OVX+E_2_ group, n=3. The *p*-value of (**A**–**C**) was determined by Student’s t test,*p<0.05,** *p*< 0.01. (**D**) Expression of PKA and p-PKA in primary cultured hypothalamic astrocytes with the intervention of different estradiol concentrations as determined by Western blotting (n = 5). (**E**) Effects of estradiol on the mRNA expression of *CYP1A1*, *CYP1A2* and *CYP1B1* gene in primary cultured hypothalamic astrocytes with the intervention of different estradiol concentrations, as compared with the control, n= 3. (**F**) Effects of PKA activator (Forskolin, 10μM, 24h) and inhibitor (H89, 30μM, 24h) on the mRNA expression of *CYP1A1*, *CYP1A2* and *CYP1B1* gene in the primary cultured hypothalamic astrocytes, as compared with the control, n=3. (**G**) Dual-label immunohistochemistry showing astrocytes (brown) and SA-β-Gal staining (blue) with the effects of 10^-6^M estradiol, together with Forskolin and H89, respectively. Black arrows represent SA-β-Gal –positive astrocytes. n=3, scale bar=50μm. The *p*-value was determined by One-way ANOVA:*p<0.05, ** *p*< 0.01. FSK, i.e. forskolin.

*CYPs* are the key enzymes in the metabolism of estradiol into hydroxyl estradiols. It is reported that estradiol activates the protein kinase A (PKA) pathway to promote neuroprogesterone synthesis in the hypothalamic astrocytes, and that PKA can enhance the expression of CYPs in tumor cell models [29, 30]. Therefore, the activation of the PKA-CYPs pathway was examined in both the in vivo animal model and the in vitro primary cultured hypothalamic astrocytes. In the young mice with regular estrous cycle, the phosphorylation of PKA in the hypothalamus was significantly higher during proestrus than during metestrus (Unpaired t test, df=10;t=10.04;*p*<0.01) (Figure 4A); Similarly, the mRNA level of the three subunits of the *CYP* gene (*CYP1A1/CYP1A2/CYP1B1*) during proestrus was also obviously higher than that during metestrus (Figure 4B). In the ovariectomized mice, the mRNA level of *CYP1A1*, *CYP1A2*, and *CYP1B1* in the OVX group was significantly lower than that in the control and OVX+E_2_ groups, while no significant difference was evident between the control and OVX+E_2_ groups (Figure 4C). In primary cultured hypothalamic astrocytes, the estradiol treatment (10^-6^M) obviously improved the phosphorylation level of PKA (one-way ANOVA effect of treatment *p*<0.01, df=3,11, F=1.297) (Figure 4D) and the mRNA expression of *CYP* subunits (Figure 4E). In contrast, no similar phenomenon was observed in the primary cultured cortical astrocytes treated with estradiol (Supplementary Figure 2A). Moreover, the cultured hypothalamic astrocytes were further respectively treated with Forskolin, an agonist for PKA, and H-89, an inhibitor for PKA. A 24-hour treatment with H-89 (10μM) decreased the expression of CYPs; however, a 24-hour treatment with Forskolin (30μM) significantly upregulated *CYPs* level (Figure 4F). These data indicate that estradiol activates the PKA pathway and then up-regulates the expression of *CYPs* in hypothalamic astrocytes.

To clarify the role of the PKA pathway in activating *CYPs* in the estradiol-induced senescence of hypothalamic astrocytes, the primary cultured hypothalamic astrocytes were respectively treated with the PKA inhibitor and agonist alone and with estradiol (10^-6^M). The interventions were divided into the following six groups: the vehicle group, 10^-6^M E_2_ group, H89 group (10μM, 24h), H89+10^-6^M E_2_ group, Forskolin group (30μM, 24h), and Forskolin+10^-6^M E_2_ group. After four consecutive 17β-estradiol interventions, no significant difference in the percentage of SA-β-Gal positive astrocytes was found among the vehicle group, H89 group and Forskolin group. Of note, the percentage of senescent astrocytes was prominently higher in Forskolin+10^-6^M E_2_ group than in Forskolin group and 10^-6^M E_2_ group. On the contrary, H89 treatment obviously decreased the E_2_-induced senescent astrocytes (one-way ANOVA effect of treatment *p*<0.01, df=5,23, F=1.668) (Figure 4G). Taken together, these results suggest that the activation of *CYPs* by PKA is a key pathway for the estradiol-induced senescence of hypothalamic astrocytes.

### Estradiol-induced senescence of hypothalamic astrocytes contributes to aging-related declines in female reproductive function

**Figure 5:**
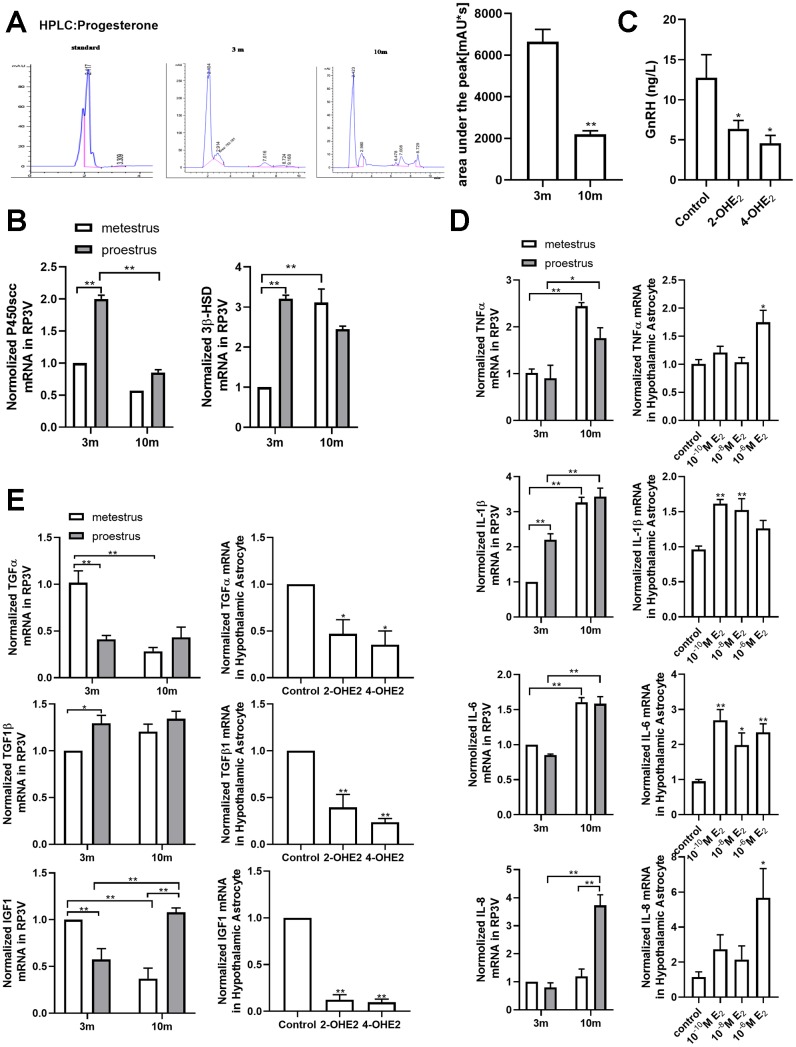
**Estradiol-induced senescence of hypothalamic astrocytes contributes to aging-related declines in female reproductive function.** (**A**) HPLC chromatogram of standard progesterone (left) and hypothalamic tissue extract of 3-month-old and 10-month-old mice. The *p*-value was determined by Student’s t test: ** *p*<0.01, n=3. (**B**) Effects of estrous cycle and age on the levels of *P450scc* and *3β-HSD* mRNA in hypothalamus as determined by qPCR. The *p*-value was determined by Two-way ANOVA: ** *p*< 0.01, n = 3. (**C**) Both 2-OHE_2_-ACM and 4-OHE_2_-ACM inhibited the secretion of GnRH from GT1-7 cells. The *p*-value was determined by One-way ANOVA: *p<0.05, n=3-4. (**D**) Effects of estrous cycle and age on the levels of *TNF-α, IL-1β, IL-6* and *IL-8* mRNA in hypothalamus as determined by qPCR. The *p*-value was determined by Two-way ANOVA: *p<0.05, ** *p*< 0.01, n = 3 (upper). Effects of estradiol on the levels of *TNF-α*, *IL-1β*, *IL-6* and *IL-8* mRNA in hypothalamic primary cultured astrocytes as determined by qPCR (n=3-6). The *p*-value was determined by One-way ANOVA:*p<0.05, ** *p*< 0.01 (blew). (**E**) Effects of estrous cycle and age on the levels of *TGF-α*, *TGF-β1*, and *IGF1* mRNA in hypothalamus as determined by qPCR (n = 3). The *p*-value was determined by Two-way ANOVA: *p<0.05, ** *p*< 0.01 (upper). Detection of *TGF-α*, *TGF-β1*, and *IGF1* mRNA levels with 2-OHE_2_ and 4-OHE_2_ intervention (n=3). The *p*-value was determined by One-way ANOVA:*p<0.05, ** *p*< 0.01.

A body of studies have suggested that hypothalamic astrocytes are involved in regulating the production of endogenous progesterone and GnRH secretion. Therefore, we detected the level of progesterone by HPLC. The results showed that the progesterone level in the hypothalamus of the young-aged mice was significantly higher than that of the 10-month-old mice (Unpaired t test, df=4; t=7.193; *p*<0.01) (Figure 5A). The key rate-limiting enzymes in progesterone synthesis, *P450scc* and *3β-HSD*, were further quantified by qPCR (Figure 5B). In 3-month-old mice, the mRNA levels of *P450scc* and *3β-HSD* were significantly higher during proestrus than during metestrus, which was not found in 10-month-old mice. Moreover, during proestrus, the mRNA expression of *P450scc* in the 10-month-old mice was obviously lower than that in 3-month-old mice. Particularly, during metestrus, the mRNA expression of *3β-HSD* in the middle-aged mice was significantly higher than that in the young ones. To investigate the effect of senescence astrocyte on GnRH secretion, the conditional medium derived from 2-OHE_2_- or 4-OHE_2_-induced aging astrocytes was collected and used to treat the hypothalamic cell line GT1-7. It showed that aging astrocyte conditioned-medium (AACM) prominently decreased the secretion of GnRH when compared with the control group (one-way ANOVA effect of treatment *p*<0.05, df=2,6, F=1.297) (Figure 5C).

The exact existence of a humoral communication between glial cells and GnRH neurons has been studied using the GT1-7 cell line and astrocytes. Studies have indicated that growth factors IGF1, TGFα and TGFβ1 released from the primary astrocyte culture participate in the cross talk between GnRH neurons and astrocytes [13–15]. qPCR results showed that during metestrus, the mRNA level of *TGFα* and *IGF1* of the middle-aged mice was obviously decreased when compared with that of the young-aged mice, with no obvious difference in *TGFβ1* expression (Figure 5E). In addition, the expression of the three genes declined significantly when the primary cultured astrocytes were treated with 2-OHE_2_ and 4-OHE_2_ while no expression difference was found when they were treated with different concentrations of estradiol. It has been reported that inflammatory pathways may impair the central regulatory networks involving GnRH neuron activity [31, 32]. However, the precise cellular and molecular effects of inflammatory factors secreted by the senescent astrocytes on GnRH neurons have not been reported yet. To gain a better insight into these regulations, the level of inflammatory cytokines was further quantified by qPCR in vivo and in vitro (Figure 5D). Not surprisingly, regardless of proestrus or metestrus, the mRNA level of inflammatory cytokines (*TNF-α*, *IL-1β* and *IL-6*) in the middle-aged mice markedly increased when compared with that of the young-aged mice. During proestrus, the mRNA expression of IL-8 in the middle-aged mice was significantly higher than that the in young-aged mice. Interestingly, repeated 17β-estradiol treatments significantly enhanced the mRNA level of *TNF-α*, *IL-1β*, *IL-6* and *IL-8* in the cultured hypothalamic astrocytes. Together, these data suggest that the reduction of progesterone and neurotrophic factors, together with the elevation of inflammatory factors in senescent astrocytes, contributes to aging-related declines in female reproductive function.

## DISCUSSION

This research found that astrocytes within the hypothalamic RP3V, not in the cortex and hippocampus, accumulate senescence-related markers with increasing age; that this cellular senescence is closely related to ovarian estradiol and compromises the regulation of progesterone synthesis and GnRH secretion. The molecular mechanism revealed that the PKA-CYPs signaling metabolized intracellular estradiol into 2-OHE_2_ and 4-OHE_2_, which were involved in the astrocyte senescence within RP3V.

It has been reported that hypothalamic astrocytes facilitate the estrogen positive feedback [7, 8, 16, 33]. The current study confirms the relationship between the estrogen positive feedback and the aging of hypothalamic astrocytes from the following three aspects. The age-dependent changes of the senescent markers of astrocytes within RP3V in intact female mice were investigated (Supplementary Figure 1A). We found that the number of peroxidase staining and SA-β-Gal-positive astrocytes increased gradually from 3 months to 9 months of age, while almost no senescent astrocytes were observed at 1 month and 2 months of age. In rodents, reproductive function is fully developed at 3 months old, accompanied by regular estrous cycles. Therefore, we also tested the effect of ovarian-derived estradiol on the senescence of hypothalamic astrocytes. In the current study, the senescent characteristics of astrocytes in RP3V of the ovariectomized 3-month-old female mice were significantly reduced (Figure 2A, 2B). In addition, to mimic the positive feedback effect of estradiol in the body, the primary cultured hypothalamic astrocytes were repeatedly interfered with different concentrations of estradiol (10^-10^M, 10^-8^M, and 10^-6^M), in which 10^-6^M estradiol for 48h has been demonstrated to induce progesterone secretion in hypothalamic astrocytes [6] and 10^-10^M estradiol is equivalent to the basic estradiol level in the normal cycle [34, 35]. As we expected, four consecutive estradiol interventions induced the senescence of hypothalamic astrocytes (Figure 2C–2E) but the phenomenon was not found in the cultured astrocytes from the cortex (Supplementary Figure 1C–1D). These results suggest that ovarian estradiol is closely related to the senescence of hypothalamic astrocytes, which is consistent with previous reports [36, 37].

Estradiol promotes the synthesis of progesterone in astrocytes by activating the cAMP/PKA pathway. Consistent with literature reports [6, 8, 38], compared with that during metestrus, the phosphorylation of PKA and mRNA level of *P450scc* and *3β-HSD* during proestrus significantly increased in the 3-month-old mice (Figures 4A and 5B). Therefore, we speculate that the PKA-CYPs pathway is involved in estradiol-induced senescence of hypothalamic astrocytes and put it to verification. In further experiments, estradiol significantly upregulated the phosphorylation of PKA and mRNA level of *CYP*s subunits in scenarios both in vivo (Figure 4A–4C) and in vitro (Figure 4D–4E). Moreover, the metabolization of estradiol into catechol estradiols by *CYPs* promoted oxidative damage to the hypothalamus. We found that 2-OHE_2_ and 4-OHE_2_ were present in the hypothalamus of the 10-month-old mice and that a low dose of 2-OHE_2_ or 4-OHE_2_ induced the senescence of cultured hypothalamic astrocytes (Figure 3). These findings are in line with the conclusion of the damage of catechol estradiols to DNA [27, 28]. Still, the regulation of PKA activity affected the estradiol-induced senescence of hypothalamic astrocytes (Figure 4G). In this study, we tried to use CYPs inhibitors to investigate whether it can inhibit the estradiol-induced astrocyte senescence. Unfortunately, CYPs are composed of multiple subtypes, and commercial CYPs inhibitors are currently not effective in inhibiting all subtypes. We expect to use CYPs blockers to prevent the senescence of hypothalamic astrocytes in the future.

Within more than two decades, many studies have shown that estradiol has a clear neuroprotective effect on AD, PD and stroke et al [39–42]. Our current conclusions concerning estradiol in the cortex do not contradict previous reports. In our study, ovarian estradiol selectively induced the senescence of hypothalamic astrocytes, not those in the hippocampus or cortex. The activation of the PKA pathway by estradiol not only mediates the process of progesterone synthesis but also up-regulates the activation of the CYPs pathway, promoting the catechin metabolism of estradiol. Of note, the activation of the PKA-CYPs pathway has not been found in the cortex during positive feedback effect of estradiol during metestrus and proestrus period (Supplementary Figure 2B, 2C). Further experimentation is needed to explore the underlying reasons for the differential activation of the PKA pathway in astrocytes from distinct brain regions.

What interests us most is the functional changes that may result from the senescent astrocytes. As expected, in the middle-aged mice, not only the level of progesterone but also the key enzyme of progesterone synthesis (P450scc) were significantly lower than those of the young mice. Another enzyme of progesterone synthesis (*3β-HSD*) was up-regulated instead, which may be a consequence of estradiol treatment [43]. Unfortunately, we have not been able to detect directly the progesterone level in the estradiol-induced senescent astrocytes, due to technical limitations, and used HPLC instead. When GT1-7 was incubated with the conditional culture medium of astrocytes (CCMA) that were pretreated by catechin estradiols, the level of GnRH secreted by GT1-7 was obviously reduced (Figure 5C). However, the pretreatment of CCMAs with the different concentrations of estradiol did not cut down the GnRH level (data not shown). We speculate that the degree of estradiol-induced senescence in astrocytes is not as serious as that by catechin estradiols (Supplementary Figure 3A). Similarly, we only detected a decrease of growth factors, *TNF-α* and *IGF1*, both in animal models of different ages and the cellular model receiving 2-OHE_2_ and 4-OHE_2_ interventions, but not in the cellular model receiving estradiol intervention. Studies have shown that a high expression of *IL-6* and *IL-1β* can inhibit the secretion of GnRH in the hypothalamus, which in turn affects the reproductive function [32, 44]. Our study found that, regardless of proestrus or metestrus, the transcription levels of *IL-1β*, *TNF-α*, and *IL-6* were significantly higher in 10-month-old mice than in their 3-month-old counterparts, respectively. These data suggest that the aging of astrocytes in the hypothalamus is implicated in the decline of GnRH neuronal activation and GnRH release.

**Figure 6:**
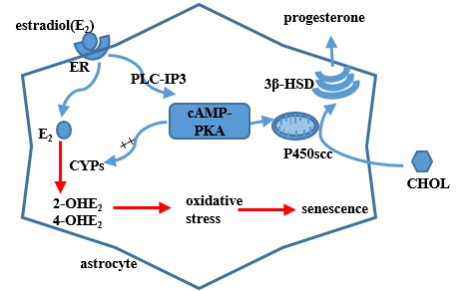
**A model of estradiol action on hypothalamic astrocytes involving the progesterone production on the basis of present results and previous findings.** Circulating estradiol acts on astrocytes that regulate progesterone (PROG) synthesis to activate the cAMP-PKA pathway. PKA can phosphorylate cytochrome P450scc and 3β-HSD, which regulate PROG production. The activated PKA pathway can also increase the expression of *CYPs* genes, which participate in the process of estradiol metabolism. Metabolic products of estradiol, 2-OHE_2_ and 4-OHE_2_, exert strong oxidative stress on astrocytes. ER, i.e. estrogen receptor; CHOL, i.e.cholesterol.

In summary, this study shows that astrocytes within the hypothalamic RP3V accumulate senescence-related markers with increasing age, accompanied by a decreased progesterone production, reduced ability of GnRH neurons to secret GnRH, decreased production of growth factors, and increased levels of pro-inflammatory cytokines. The underlying mechanism involves the activation of PKA-CYPs pathway by ovarian estradiol, promoting the metabolization of estradiol into 2-OHE_2_ and 4-OHE_2_ and accelerating the astrocyte senescence within RP3V (Figure 6). The findings confirm that ovarian estradiol induces the senescence of hypothalamic astrocytes. The senescent astrocytes compromise the regulation of progesterone synthesis and GnRH secretion, which may contribute to aging-related declines in female reproductive function.

## MATERIALS AND METHODS

### Animals and treatment protocols

All C57BL/6J female mice were purchased from Slack Animal Co (Shanghai, China). Experimental mice were housed (≤ 5 animals/cage) in a pathogen-free colony (IVC system, Tecniplast, Italy) and allowed free access to food and water. The colony was maintained under a 12-h light/12-h dark cycle with the temperature set at 24 °C. All protocols and procedures used in these studies were approved by the Institutional Animal Care and Use Committee of Fujian Medical University and in compliance with NIH’s Guidelines for the Care and Use of Laboratory Animals (NIH Publication No. 85-23 Rev. 1985). Estrous cycles of the young-aged mice (3–4 months old) and middle-aged mice (9–10 months old) were monitored by daily vaginal smears. In this study only mice having at least two consecutive cycles of a regular 4–5 day estrous period were included. The selected mice received, under anesthesia, a bilateral ovariectomy (OVX) or OVX plus a pellet containing 0.25mg of 17β-estradiol (OVX+E_2_, Innovative Research of America, #NE-121), which undergoes a 90-day continual release and was subcutaneously implanted in the posterior neck. The OVX+E_2_ group received another embedded 17β-estradiol sustained-release tablets 90 days later. Animals were transported to the operating room for tissue harvest at 9 months old.

### Transmission electron microscopy

The mice were anesthetized with 10% chloral hydrate and injected with normal saline, and their brains were immediately removed. The hypothalamic area was dissected (1×1×1 mm), immersed in 2.5% glutaraldehyde buffer, fixed in 1% tetroxide, dehydrated in the gradient concentration ethanol, and finally embedded in Epon812. The targeted subregion was observed under a transmission electron microscope (EM208, Royal Philips Electronics, Netherlands).

### Hypothalamus dissection and qPCR

The experiments were performed as to previously reported [4]. Briefly, animals were anaesthetized and brains were collected. The preoptic area anterior hypothalamus (POA-AH) for biochemical experiment. For POA-AH, the caudal border was made by a coronal cut just posterior to the entry point of the optic chiasm; the rostral border was exactly 3mm anterior. This coronal section (3 mm thick) was laid rostral side up on a chilled glass plate, then an isosceles triangle-shaped cut was made with its apex just under the midline of the corpus callosum and the 2 legs passing through the anterior commissure. Total RNA was extracted from the dissected hypothalamus and reversely transcribed into cDNA. The primer sets were presented in the Supplementary Table 1. Each reaction was performed in triplicate. Fold changes were calculated by 2^-ΔΔt^, where ΔCt =Ct (target gene) - Ct (actin/GAPDH) and Δ(ΔCt) =ΔCt (experimental groups) –mean ΔCt.

### Senescence-associated β-galactosidase (SA-β-Gal) assay combined with immunohistochemistry

The experiments were performed as to previously reported [4]. Briefly, the dissected brains were post-fixed in 4 % paraformaldehyde, dehydrolyzed in 30 % sucrose, and then sliced into sections (40μm). For SA-β-Gal staining, sections were immersed in a SA-β-Gal staining solution. After incubation at 37 °C in the dark overnight, sections were washed and treated with 3 % H_2_O_2_ for 10 min, and then washed. They were next blocked in a blocking buffer for 60 min, then incubated with Anti-Iba1or anti-GFAP (Supplementary Table 2) in TBS for 24 h. They were then incubated with biotinylated second antibody at RT for 90 min. After being washed in TBS, these sections were further incubated in Vector Elite avid in peroxidase at RT for 1h. Sections were stained with DAB for 1–10 min, then cover-slipped with a permanent mounting medium, and detected under a microscope.

### Peroxidase assay combined with immunohisto-chemistry

The sections were washed with TBS and then developed directly with DAB for 10 min. After another TBS wash, the brain sections were examined by immunohistochemistry.

### Immunofluorescence

The experiments were performed as to previously reported [4]. Briefly, sections were washed and then blocked at RT for 1h. To detect astrocytes, the sections were incubated at 4 °C for 24 h with primary antibodies in TBS: polyclonal chicken anti-GFAP, polyclonal rabbit anti-GS, polyclonal rabbit anti-P16, and monoclonal mouse anti-γH2AX (Supplementary Table 2). The sections were then washed and incubated at RT for 1 h in a mixture of secondary antibodies. Afterwards, the sections were washed, mounted on glass slides, and then cover-slipped with prolong Gold anti-fade reagent.

### Hypothalamic primary astrocyte cultures and conditioned medium

The astrocytes were obtained from the brains of neonatal mice (d1-2, C57BL/6J). Hypothalamus and cortices were dissected, freed of meninges in an ice-cold salt ion buffer. Digestive enzyme was compounded using Papain Suspension (Worthington, Lakewood, NJ) and salt ion buffer. The separated brain tissue was placed in the filtered digestive enzyme and dissolved in an incubator with 5% CO_2_ at 37°C for 20min. Then, enzyme activity was blocked by an excess of fetal bovine serum (FBS, HyClone, Utah, USA). All of the enzymes were removed and tissues were mechanically dissociated in a DME/F12 medium (HyClone, Utah, USA) containing 10% FBS and 1% penicillin-streptomycin (HyClone, Utah, USA). After mechanical dissociation, cells were seeded and maintained in an incubator with 5% CO_2_ at 37°C for 2 weeks in DME/F-12 containing 10% FBS and 1% penicillin-streptomycin in poly-L-lysine coated 6-well or 24-well plates. The culture medium was refreshed twice a week. The purity of astrocytes was about 95% of GFAP-positive cells.

Prior to drug treatment, all astrocytes were subjected to steroid starvation for 24 hours in DME/F12 (supplemented with 5% of fetal bovine serum separated by charcoal; Sigma-Aldrich, F6765, St. Louis, MO). After steroid-starvation, some cultures were respectively treated for 48h with 17β-estradiol (10^-10^M, 10^-8^M, 10^-6^M; Sigma-Aldrich, E2758, St. Louis, MO), Forskolin (10μM, 24h), H89 (30μM, 24h), 17β-estradiol (10^-6^M) + Forskolin, 17β-estradiol (10^-6^M) + H89, or estradiol-free DME/F12 culture media. The process of steroid starvation and drug treatment was repeated 4 times. Other cultures were respectively treated with 2-OHE_2_ (20nM) and 4-OHE_2_ (20nM) for 24h before the medium refreshment. After 36h, the collected media were cultured with the GT1-7 lines for 48h.

### Sample preparation and HPLC analysis

The weighed sample was ground and suspended in a mixture of water and methanol (1:1). The sample was homogenized and then centrifuged by high-speed centrifugation in a vacuum. The residue was dissolved in water and methanol (1:1), filtered through a 0.2μ ultracentrifuge filter (Merck Millipore, MA, USA) and prepared for HPLC analysis. The process was repeated in duplicate during HPLC analysis. Pure standards were used to optimize the HPLC conditions prior to sample analysis. HPLC was performed by Shanghai Institute of Organic Chemistry, Chinese Academy of Science.

### Statistical analysis

Data were presented as means ± standard error (SEM) of a percent relative ratio. Statistical comparisons between 2 independent groups were analyzed by the unpaired Student’s t-test; comparisons among 3 or more independent groups, were by a one-way analysis of variance (ANOVA), together with Kruskal-Wallis test where appropriate. Data were processed using GraphPad Software. *p* < 0.05 was considered statistically significant.

## Supplementary Material

Supplementary Figures

Supplementary Tables
