# Supplementary material for: Estradiol-induced senescence of hypothalamic astrocytes contributes to aging-related reproductive function declines in female mice
Source: Aging (Albany NY). 2020 Apr 7;12(7):6089–108. doi: 10.18632/aging.103008 (PMC7185128; doi:10.18632/aging.103008)
Supplement: Supplementary Figures [file aging-12-103008-s002..pdf]

SUPPLEMENTARY FIGURES

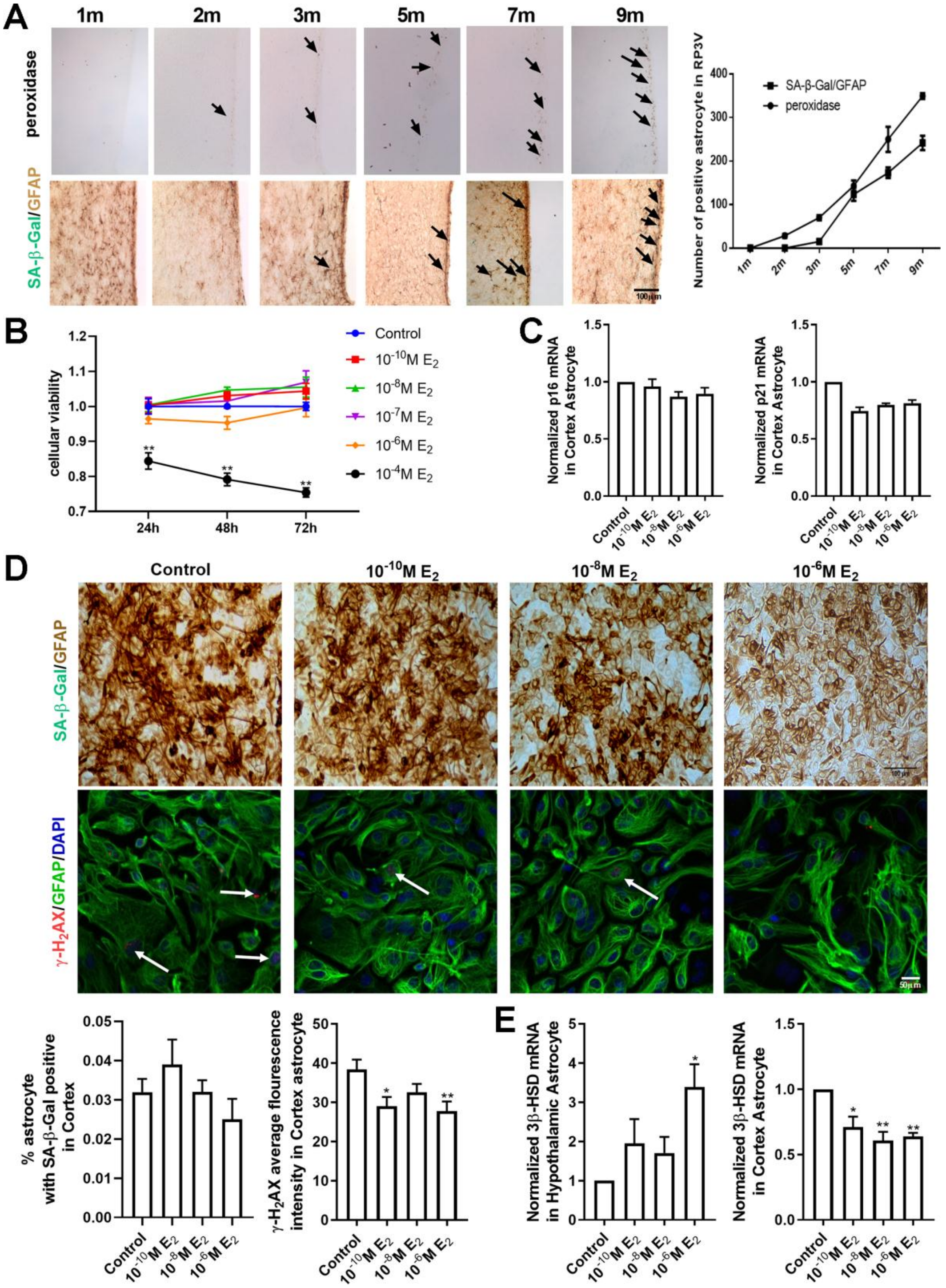

Supplementary Figure 1. (**A**) Peroxidase staining (brown) in the astrocyte of RP3V region in mice at different ages (up pictures), black arrows representing peroxidase. GFAP (black) and peroxidase (brown) double staining in the astrocyte of the RP3V region of the hypothalamus in mice at different ages (down pictures), black arrows representing peroxidase–positive astrocytes. n=5, Scale bar =100 µm. (**B**) CCK8 assay of astrocyte cultured with different estradiol concentrations.10^{-4}M E_2 inhibited cell growth at 24h, 48h and 72h, n=10,**p<0.01. (**C**) Detection of the mRNA levels of *p16* and *p21* under different estradiol concentrations in the cortex astrocyte(n = 3-4). (**D**) Dual-label immunohistochemistry showing astrocytes (brown) and SA-β-Gal staining(blue) with three different estradiol concentrations(10^{-10}M, 10^{-8}M, 10^{-6}M),scale bar=100µm; dual-label immunofluorescent showing astrocyte(green) and γ-H2AX (red) with

different estradiol concentrations, white arrows representing γ-H2AX–positive astrocytes. Estradiol did not affect the percentage of the SA-β-Gal positive cells in different estradiol concentrations. Estradiol decreased the percentage of γ-H2AX–positive astrocytes in different estradiol concentrations (**p<0.01). Estradiol decreased the average fluorescence intensity in different estradiol concentrations, (*p<0.05 for 10^{-10}M and **p<0.01 for 10^{-6}M). Scale bar=50µm. n=3. (**E**) Detection of *3β-HSD* mRNA levels in astrocyte from hypothalamus and cortex under different estradiol concentrations. Only 10^{-6}M estradiol increased the expression of *3β-HSD* gene in hypothalamic astrocytes. Estradiol decreased the expression of *3β-HSD* with different estradiol concentrations in cortex astrocytes (n = 3-4). The p-value was determined by One-way ANOVA:*p<0.05, ** p< 0.01.

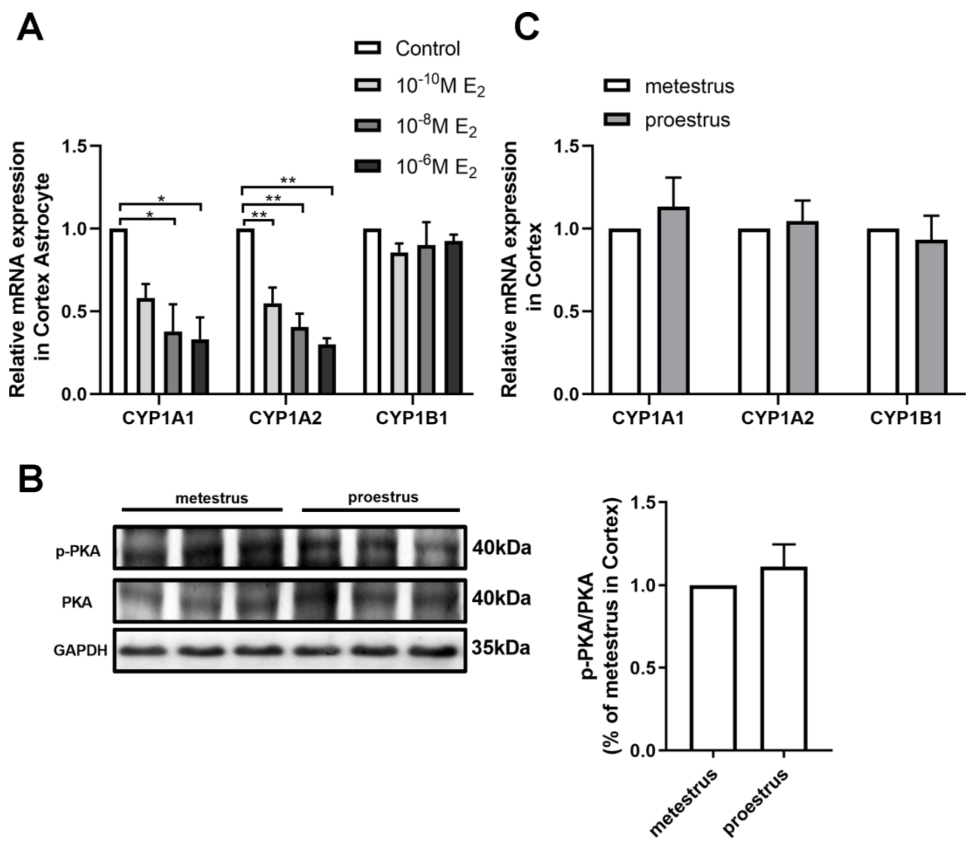

Supplementary Figure 2. (**A**) Effects of estrous cycle on the mRNA expression of *CYP1A1*, *CYP1A2* and *CYP1B1* gene in the cortex. The *p*-value was determined by One-way ANOVA, ns, metestrus compared with proestrus (n=3). (**B**) Expression of PKA and p-PKA in the cortex of proestrus and metestrus at 3 months of age. Western blot analysis was normalized by GAPDH as the loading control. ns, metestrus compared with proestrus (n = 6). (**C**) Detection of *CYPs* mRNA levels under different estradiol concentrations in the cortex astrocyte (n = 3-4). The *p*-value of (a) and (b) was determined by Student's t test.

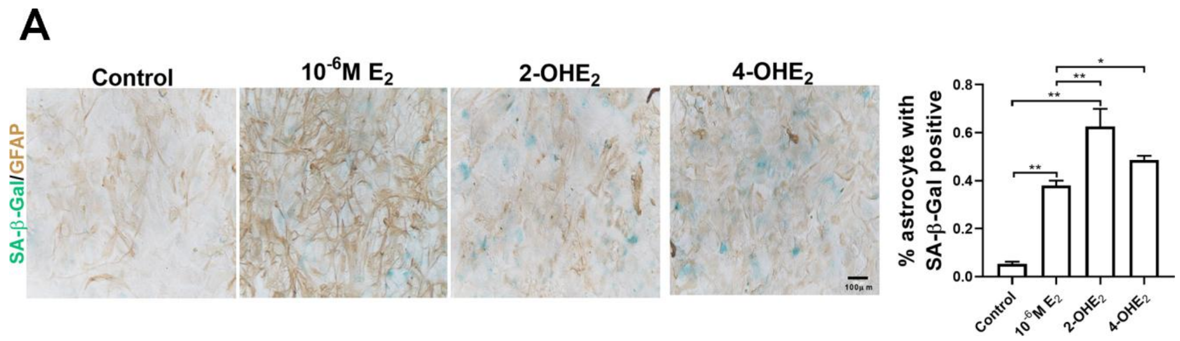

Supplementary Figure 3. (**A**) Dual-label immunohistochemistry showing astrocytes (brown) and SA-β-Gal staining (blue) with treatment of 10^{-6}M estradiol, 2-OHE_2 and 4-OHE_2. Black arrows represent SA-β-Gal–positive astrocytes. The number of SA-β-gal-positive cells in 10^{-6}M E_2 group was much smaller than that in 2-OHE_2 and 4-OHE_2 groups, respectively (n=5). The *p*-value was determined by One-way ANOVA: ** $p<0.01$. Scale bar=100µm.
