# Supplementary material for: Estradiol-induced senescence of hypothalamic astrocytes contributes to aging-related reproductive function declines in female mice
Source: Aging (Albany NY). 2020 Apr 7;12(7):6089–108. doi: 10.18632/aging.103008 (PMC7185128; doi:10.18632/aging.103008)
Supplement: Supplementary Tables [file aging-12-103008-s001..pdf]

SUPPLEMENTARY TABLES

Supplementary Table 1. Primer sequences.

Gene	Primer sequences
CYP1A1	Forward, 5'GGTTAACCATGACCGGGAACT3'
	Reverse, 5'TGCCCAAACCAAAGAGAGTGA3'
CYP1A2	Forward, 5'ACATTCCCAAGGAGCGCTGTATCT3'
	Reverse, 5'GTCGATGGCCGAGTTGTTATTGGT3'
CYP1B1	Forward, 5'GTGGCTGCTCATCCTCTTTACC3'
	Reverse, 5'CCCACAACCTGGTCCAACTC3'
P450ssc	Forward, 5'GACCAAGTTCAGCCTCATCC3'
	Reverse, 5'CTCCAGCCTTCAGTTCACAG3'
3β-HSD	Forward, 5'TTGTCATCCACACTGCTGCT3'
	Reverse, 5'TGGCACACTTGCTTGAACAC3'
p16	Forward, 5'CCCAACGCCCCGAACT3'
	Reverse, 5'GCAGAAGAGCTGCTACGTGAA3'
p21	Forward, 5'GGCAGACCAGCCTGACAGAT3'
	Reverse, 5'TTCAGGGTTTTCTCTTGCAGAAG3'
TNF-α	Forward, 5'GACGTGGAACTGGCAGAAGAG3'
	Reverse, 5'TTGGTGGTTTGTGAGTGTGAG3'
IL-1β	Forward, 5'TTCAGGCAGGCAGTATCACTC3'
	Reverse, 5'GAAGGTCCACGGGAAAGACAC3'
IL-6	Forward, 5'TAGTCCTTCCTACCCCAATTTCC3'
	Reverse, 5'TTGGTCCTTAGCCACTCCTTC3'
IL-8	Forward, 5'TCGAGACCATTTACTGCAACAG3'
	Reverse, 5'CATTGCCGGTGGAAATTCCTT3'
TGF-α	Forward, 5'GGAACCTGCCGGTTTTTGG3'
	Reverse, 5'CACAGCGAACACCCACGTA3'
TGF-β1	Forward, 5'CTCCCGTGGCTTCTAGTGC3'
	Reverse, 5'GCCTTAGTTTGGACAGGATCTG3'
IGF-1	Forward, 5'AAATCAGCAGCCTTCCAACTC3'
	Reverse, 5'GCACTTCCTCTACTTGTGTTCTT3'
GAPDH	Forward, 5'CAGTGGCAAAGTGGAGATTGTTG3'
	Reverse, 5'CTCGCTCCTGGAAGATGGTGAT3'

Supplementary Table 2. Antibodies used in the study.

Antibodies	Manufacturer	Catalogue No.	Dilution
Anti-Iba1	Wako	019-19741	1:1000 for IHC
Anti- GFAP	Covance	SMI-21R-100	1:1000for IHC
Anti- GFAP	Covance	PCK-591P-100	1:2000 for IF
Anti-GS	MilliporeSigma	G2781	1:5000 for IF
Anti-P16	Santa Cruz Biotechnology	sc-1207	1:1000 for IF
Anti-γH2AX	Abcam	ab26350	1:1000 for IF
Anti-PKA	Cell Signaling Technology	#5842	1:1000 for WB
Anti-p-PKA	Cell Signaling Technology	#5661	1:1000 for WB
Anti-GAPDH	Abcam	ab127428	1:5000 for WB
